# Fretting Corrosion Behavior of Multilayer Structure on Nitrided 2.25Cr-1Mo Steel in 723 K Liquid Sodium

**DOI:** 10.3390/ma19132815

**Published:** 2026-07-02

**Authors:** Xudong Chen, Weifei Hu, Fuguo Chen, Liwen Wang, Jun Wang

**Affiliations:** 1 School of Mechanical Engineering, Zhejiang University, Hangzhou 310058, China; 2 Dongfang Electric Yangtze River Delta (Hangzhou) Innovation Institute Co., Ltd., Hangzhou 311112, China; 3 College of Aviation Engineering, Civil Aviation Flight University of China, Guanghan 618307, China

**Keywords:** 2.25Cr-1Mo steel, QPQ, fretting corrosion, deterioration mechanism, liquid sodium

## Abstract

**Highlights:**

- A multilayer structure composed of an oxide layer, nitride layer, and diffusion layer was successfully fabricated on the surface of 2.25Cr-1Mo steel, significantly improving its cross-sectional hardness.
- Higher nitriding temperature (QPQ 590) promotes the formation of a thicker modified layer, it concurrently leads to the premature generation of micro-cracks within the compound layer.
- Under high-temperature sodium environments, the synergistic interaction of mechanical friction, chemical corrosion, and thermal effects accelerates material loss at the friction interface. Microcracks generated during fabrication significantly expedite this process.
- Strict control over surface and interfacial microcracks during the surface modification of 2.25Cr-1Mo steel is critical to enhancing its long-term service performance.

**Abstract:**

In this study, multilayer modified structures were fabricated on the surface of nuclear-grade 2.25Cr–1Mo steel via salt bath nitriding at different temperatures. Fretting corrosion tests were subsequently conducted in liquid sodium at 723 K. The results indicate that the multilayer structures formed by salt bath nitriding effectively enhance the cross-sectional hardness and improve the wear resistance of the substrate. However, after prolonged exposure to liquid sodium at 723 K, these multilayer structures undergo failure, primarily manifesting as cracking, spalling, and corrosion micropores. Material degradation of the nitrided steel is governed by the synergistic effects of tribological removal, chemical corrosion, and thermal acceleration. Notably, the QPQ 550 treatment, featuring a thinner compound layer, exhibited superior tribological performance during extended testing. This is attributed to the fact that while a higher salt bath nitriding temperature (QPQ 590) yields a thicker multilayer structure, it simultaneously induces premature failure—characterized by microcracking and the formation of corrosive channels—which ultimately compromises the wear performance.

## 1. Introduction

Countries around the world have accelerated research on fourth-generation fast neutron reactors due to the increasing energy demand and the lack of uranium resources [1,2,3,4]. Among them, the sodium-cooled fast reactor with the most commercial value has great advantages in safety and reliability, reducing environmental burden, efficient utilization of resources, and economic competitiveness [5,6]. Compared with other coolants, liquid sodium has a higher heat transfer coefficient, heat capacity, and a wider liquid temperature range (98–863 °C) and can achieve a high power density core at atmospheric pressure, which is friendly to the reactor structural materials [7,8]. However, sodium also brings some hidden dangers, such as alloy elements and impurities (O, C) in sodium, which will corrode the structural materials, and the liquid sodium will react violently in contact with water or water vapor [9,10]. In a steam generator (SG) of SFR, the high-pressure water and water vapor, and the low-pressure liquid sodium flow inside and outside the heat transfer tube, respectively. The temperature difference between the two-phase flow and the pressure difference inside and outside the tube will cause nonlinear vibration between the tube and its support plate, which will lead to fretting wear of the heat exchange tube [11,12]. After long-term service, the heat transfer tube may rupture, resulting in a violent reaction between sodium and water or water vapor (generating a lot of heat, strong corrosive NaOH, and shock waves) [10,13,14]. This not only causes greater vibration and wear of the heat transfer tubes but also affects other surrounding heat exchange tubes, resulting in a chain reaction, which endangers the safety of the reactor. The tribo-corrosion of heat transfer tubes is a complex interfacial reaction, including material removal by friction force, deterioration of material properties due to thermal effects, chemical corrosion, and nuclear radiation. Studying the fretting corrosion behavior and interfacial failure mechanism of heat transfer tubes is crucial for the structural safety of SGs [15].

At present, the 2.25Cr-1Mo, low-carbon alloy steel with excellent resistance to thermal stress and swelling, is the commonly used material for the heat exchange tube of the SG in SFR [16,17,18]. The microstructure [19,20], welding [21], high-temperature creep [22,23], service-exposed life [24,25,26], friction [27,28,29,30], and other properties of 2.25Cr-1Mo have been widely studied. The excellent properties of 2.25Cr-1Mo steel come from the dispersed fine chromium (Cr) and fine molybdenum (Mo) carbides [31]. However, these fine carbides will gradually grow under the action of long-term high temperatures, which will degrade the material properties [32]. Moreover, both the hardness and the friction performance of 2.25Cr-1Mo steel are not good. A feasible choice to strengthen the surface of 2.25Cr-1Mo steel is surface modification technology. Our previous study has found that the Quench–Polish–Quench complex salt bath (QPQ) treatment can improve the friction properties of 2.25Cr-1Mo steel in liquid sodium [33]. However, the fretting corrosion mechanism of the multilayer structure prepared by QPQ on 2.25Cr-1Mo steel under high-temperature liquid sodium has not been clearly explained. Therefore, this paper adopts the multi-layer structures of different thicknesses prepared by QPQ. Through a longer fretting corrosion test, the deterioration mechanism of nitrided 2.25Cr-1Mo steel in liquid sodium at 723 K and the fretting corrosion mechanism of the friction interface were further discussed.

## 2. Materials and Methods

### 2.1. Materials and Sample

In this study, the nuclear grade 2.25Cr-1Mo steel (Fe bal, Cr 1.9–2.6, Mo 0.87–1.13, Mn 0.3–0.6, Si ≤ 0.50, S ≤ 0.025, P ≤ 0.025, C 0.05–0.15 (wt%)) was machined to a sample with a length of 55 mm and a diameter of 16 mm and a wall thickness of 1.2 mm. The Gr5C12 steel (Fe bal, Cr 4–6, Mo 0.45–0.65, Mn 0.3–0.6, Si ≤ 0.50, S ≤ 0.025, P ≤ 0.025, C ≤ 0.15 (wt%)) with a length of 20 mm and a diameter of 10 mm is used as a tribo-pair. The surface hardness of the 2.25Cr-1Mo steel is 183.5 HV0.5 (RT), with the average roughness (Ra) of the specimen surface ranging from 0.12 to 0.13 μm, and that of the Gr5C12 steel ranging from 0.11 to 0.13 μm. The 2.25Cr-1Mo steel specimen was in orthogonal contact with the tribol-pair (cylinder–cylinder). They are all provided by the Central Research Academy of Dongfang Electric Corporation (Chengdu, China). The prepared 2.25Cr-1Mo steel was subjected to QPQ treatment at different salt bath temperatures. The samples were first degreased with kerosene and subsequently preheated in air at 400 °C for 30 min to eliminate surface moisture. Following this, they were immersed in a salt bath nitriding furnace and held at 550 °C for 90 min to form a high-performance nitrided layer on the surface. The samples were then cooled to 400 °C and subjected to salt bath oxidation for 30 min to decompose residual cyanide radicals and generate an oxide film, thereby enhancing corrosion resistance. Next, the oxidized specimens were polished for 60 min to remove coarse nitride and oxide particles and reduce surface roughness. Subsequently, a secondary salt bath oxidation was conducted at 400 °C for 15 min to develop a dense black oxide layer. Finally, the samples were thoroughly rinsed with water. Throughout the salt bath process, the CNO^−^ content in the nitriding salt was maintained within the range of 32–33% [33]. The nitrided samples with the salt bath temperature of 550 and 590 °C were named QPQ 550 and QPQ 590, respectively.

### 2.2. Fretting Corrosion Test

A self-made fretting corrosion test rig was used for the experiments. It can achieve a tangential fretting wear test in a liquid sodium environment with the highest temperature of 500 °C, the maximum load of 50 N, the maximum displacement amplitude of 200 μm, and the maximum motion frequency of 20 Hz. The detailed introduction and operation process can be referred to in our previous research [33,34,35]. In this study, according to the actual working conditions of the heat transfer tube [8,36] and the other studies [11,37,38], the test parameters are set as follows: the sodium temperature is 723 K, the normal load is 20 N, the displacement amplitude is 50 μm, and the frequency is 5 Hz designed to simulate the in-service conditions of heat exchanger tubes. To obtain longer-term results and better analyze the surface deterioration and the interface tribo-corrosion mechanism of nitrided 2.25 steel subjected to nitriding at two different temperatures in liquid sodium, two cycles of 2 × 10^5^ and 2 × 10^6^ were set. Through the symmetrical module design, two groups of samples under the same test parameter can be tested at one time, which can save time and avoid errors caused by the operation. The tribo-pair was driven by the drive shaft to perform linear reciprocating motion, with the fretting direction aligned with the radial direction of the specimen. In addition, impurities in sodium (especially O and C) are one of the main factors affecting the corrosion of structural materials. Therefore, fresh pure sodium was used for each test. To prevent air or water vapor from entering and increasing the oxygen content in the sodium, argon gas was continuously injected during the test to maintain a low-oxygen environment. After each test, the used sodium was recovered by the facility, and the testing equipment was thoroughly cleaned with anhydrous ethanol to remove any residual sodium, followed by heating and drying to eliminate moisture. Every experimental group underwent three repetitions, with the average value serving as the wear result. After the test, the volume of material transferred or lost was obtained using the special software version, and the average value was obtained by measuring it three times.

### 2.3. Microstructure and Composition Characterizations

Before the test, the microstructure and phase of the nitrided samples were characterized by a three-dimensional topography instrument (Bruker Corporation, Billerica, MA, USA), scanning electron microscope (Thermo Fisher Scientific, Waltham, MA, USA), energy dispersive spectrometer (Thermo Fisher Scientific, Waltham, MA, USA), and X-ray diffraction (XRD). The cross-sectional microstructure and hardness of the nitrided samples were analyzed by SEM and a Vickers hardness tester (Shandong Zongde Electromechanical Equipment Co., Ltd., Jinan, China). After the test, the morphology of the wear scar is mainly analyzed by a three-dimensional topography instrument, and the material transfer or loss volume is calculated. The surface-section microstructure and tribo-corrosion behavior of the wear scars and the unworn areas were characterized by SEM and EDS (in this study, the EDS measurements of nitrogen and oxygen are presented for qualitative analysis only). The phase changes on the surface of the nitrided samples after corrosion, and in the wear scar micro-area after fretting corrosion, were analyzed by XRD. The QPQ590 after 2 × 10^6^ tests was selected for TEM (Thermo Fisher Scientific, Waltham, MA, USA) analysis. The selected area electron diffraction (SAED) and high-resolution transmission electron microscopy (HRTEM) tests were carried out on the subsurface of the samples for further analysis.

## 3. Results and Discussion

### 3.1. Microstructure and Composition of the Nitrided Multilayer Structure

**Figure 1:**
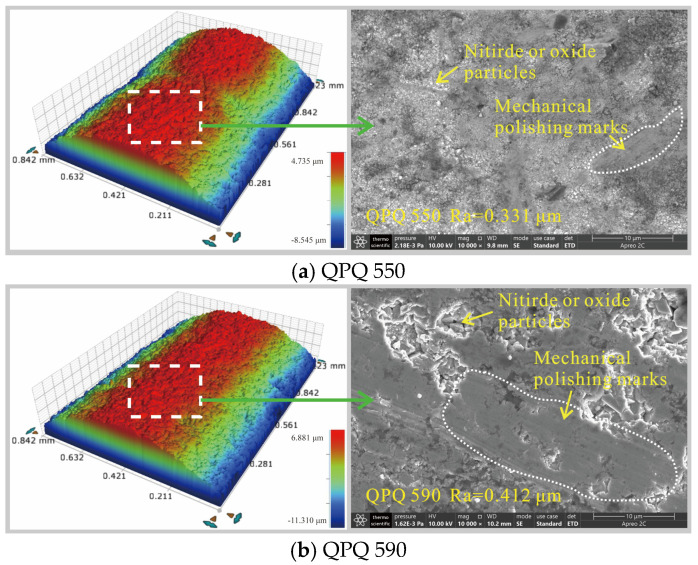
The 3D surface morphology, and SEM images of the nitrided samples.

**Figure 2:**
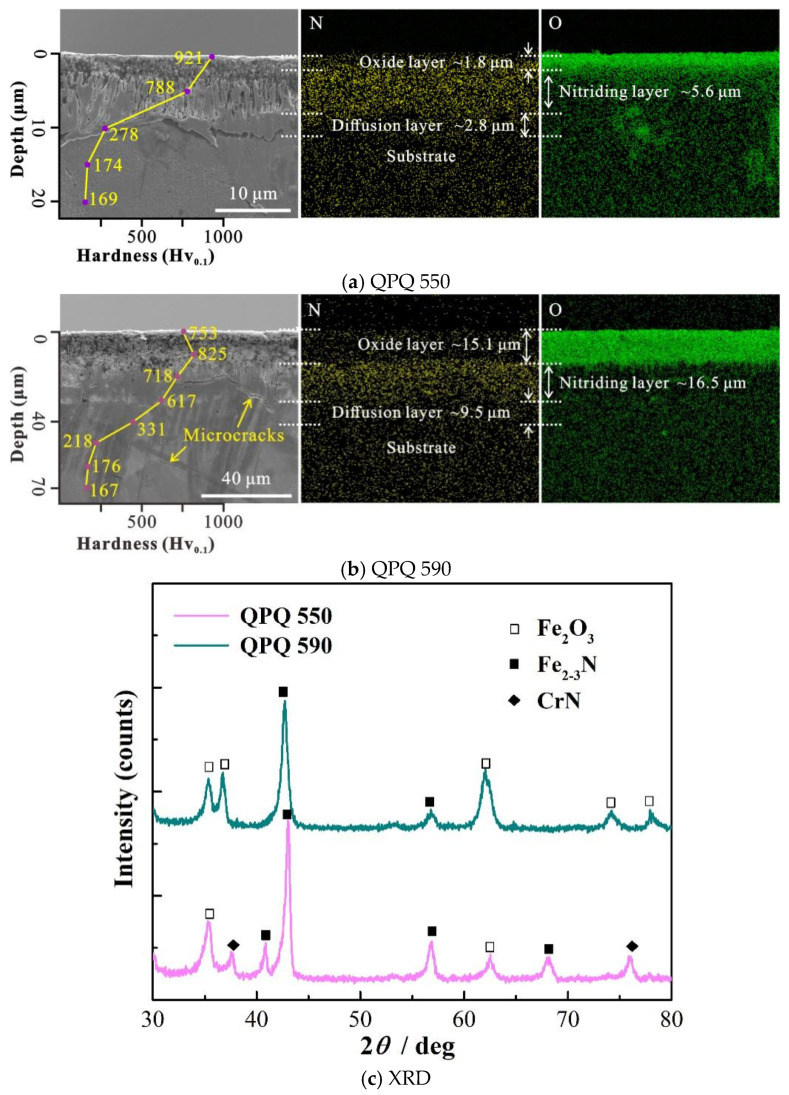
The cross-sectional SEM images, O and N distribution, hardness (**a**,**b**), and the XRD (**c**) of the nitrided samples.

Figure 1 presents the surface 3D topography and microstructure of the nitrided samples. After the QPQ complex salt bath heat treatment, the samples exhibited uneven surfaces, and the roughness of QPQ 550 and QPQ 590 were 0.331 and 0.412 μm, respectively. Some particulates (nitride or oxide) and mechanical polishing traces were found on the samples, as shown in Figure 1a,b. Figure 2 presents the microstructure, element distribution, and hardness of the cross-section of the nitrided sample. From the distribution of N and O elements, it can be found that the section of nitrided 2.25Cr-1Mo steel is a multilayer structure. The outermost layer is an O-rich oxide layer obtained by salt bath oxidation, followed by an N-rich nitride layer [39], as shown in Figure 2a,b. The N element of the diffusion layer is not enriched, but the hardness of the diffusion layer is greater than that of the substrate in terms of cross-sectional hardness.

**Figure 3:**
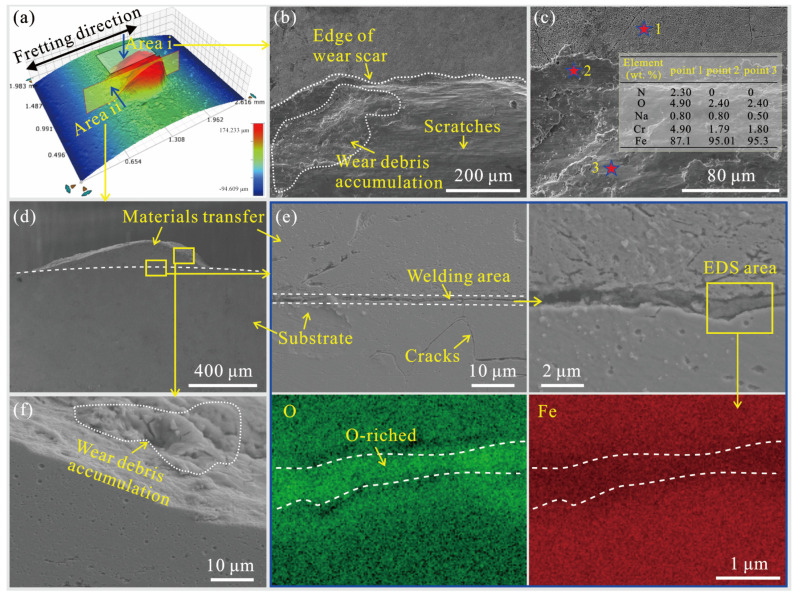
The micro-morphology analysis of the wear scar of QPQ 550, N = 2 × 10^5^: the 3D morphology (**a**); the SEM images and EDS of the worn surface (**b**,**c**), worn surface (**e**) and the cross-section (**d**,**f**).

The thickness of the multilayer structure increased with the increase in salt bath temperature [40]. The thickness of the oxide layer, nitriding layer, and diffusion layer of the QPQ 550 is about 1.8, 5.6, and 2.8 μm (Figure 2a), respectively, while QPQ 590 is 15.1, 16.5, and 9.5 μm (Figure 2b). The increase in section hardness mainly comes from the nitriding layer (about 4–5.5 times than substrate), and the strengthening depth increases with the thickness of the multilayer structure [41,42], as shown in Figure 3a,b. In addition, some microcracks were found in the multilayer structure and substrate of QPQ 590 (Figure 2b), which was caused by the excessive growth stress caused by the nitride growth and the decrease in the toughness of the multilayer structure [43,44]. It is suggested that a higher salt bath temperature will cause premature failure (microcracks) of 2.25Cr-1Mo steel [33]. The XRD patterns of the nitrided samples are shown in Figure 2c. Fe_2_O_3_, Fe_2−3_N, and CrN are the main substances obtained after nitriding [45,46,47]. The nitride peak of QPQ 550 is stronger, while the oxide peak of QPQ 590 is stronger. This may be due to the thicker loose oxide layer of QPQ 590, which also makes the surface hardness of QPQ 590 slightly lower than that of QPQ 550 (Figure 2b).

### 3.2. Fretting Corrosion Behavior in Liquid Sodium

**Figure 4:**
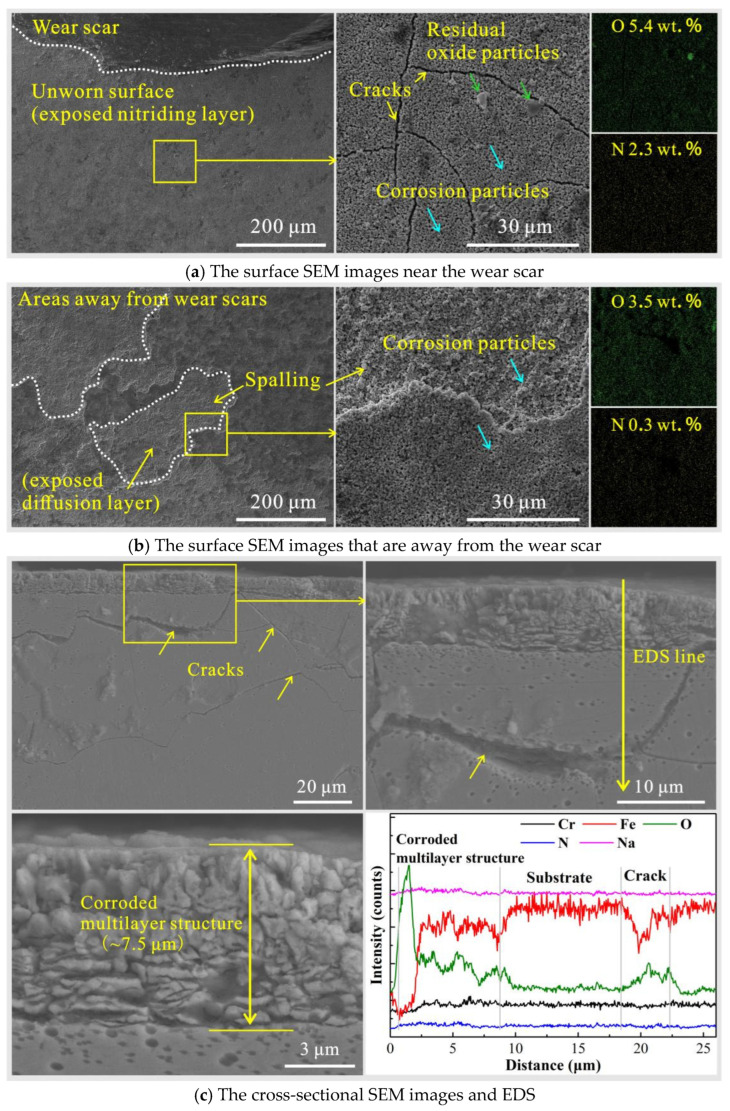
The micro-morphology analysis of the unworn areas of QPQ 550, N = 2 × 10^5^.

Figure 3 and Figure 4 show the characteristics of wear scars and unworn areas for QPQ 550 after 2 × 10^5^ cycles. The 3D morphology found that the wear scar had a “convex peak” that was significantly higher than the substrate, as shown in the red area in Figure 3a. The enlarged views of the surface and cross-section of the marked region in Figure 4a are shown in Figure 3b,c and Figure 4a–c, respectively. Some wear debris deposits and grooves parallel to the fretting direction on the worn surface (Figure 3b). Elemental analysis showed that the worn surface was mainly Fe, and a small amount of O, Cr, and a trace amount of Na, while a small amount of N existed in the unworn area (Figure 3c). Figure 3d,e finds an O-rich weld between the “convex peak” and the substrate [48]. In addition, some microcracks were also found in the substrate section (Figure 3e). This may be due to the performance degeneration of 2.25Cr-1Mo steel in a high-temperature environment [20,22]. Figure 3f also found some wear debris accumulated on the edge of the wear scar, which agrees with the results in Figure 3b.

Figure 4a shows the SEM image close to the wear scar. The outermost oxide layer was almost completely reacted with sodium and removed [36], leaving only a few oxide particles (Figure 4a). The exposed nitrided layer was corroded by sodium and produced a large number of cracks. On the surface, far from the wear scar, the nitrided layer also peeled off, and the exposed diffusion layer was corroded by sodium, resulting in a large number of corrosion products (Figure 4b). This was confirmed by elemental scan analysis, with the N content of the exposed nitrided layer slightly higher than that of the exposed diffusion layer (Figure 4a,b). The cross-section of the unworn area shows that the multilayer structure developed many cracks, and the cracks penetrated the matrix, as shown in Figure 4. Nitriding increases the hardness and brittleness of the material, but reduces the toughness of the material. After exposure to high-temperature liquid sodium, the entire multilayer structure was destroyed due to long-term thermal effects. The fractured multilayer structure accelerates sodium corrosion, and the microcracks in it become channels for rapid corrosion [35]. This was confirmed by EDS line scan results, where strong O was found in the multilayer structure and cracks (Figure 4c). After corrosion, the thickness of the multilayer structure is reduced to only about 7.5 μm. This is consistent with the results of Figure 4a,b, mainly because of the exfoliation of the oxide and nitride layers.

**Figure 5:**
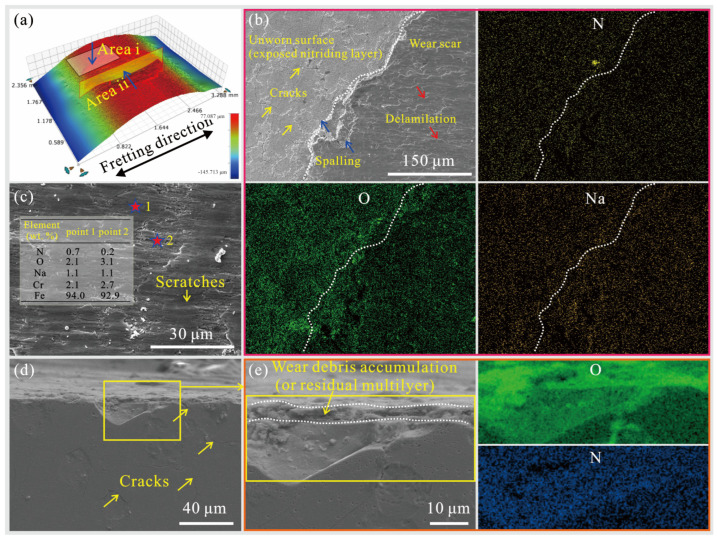
The micro-morphology analysis of the wear scar of QPQ 590, N = 2 × 10^5^: the 3D morphology (**a**); the SEM images and EDS of the worn surface (**b**,**c**) and cross-section (**d**,**e**).

**Figure 6:**
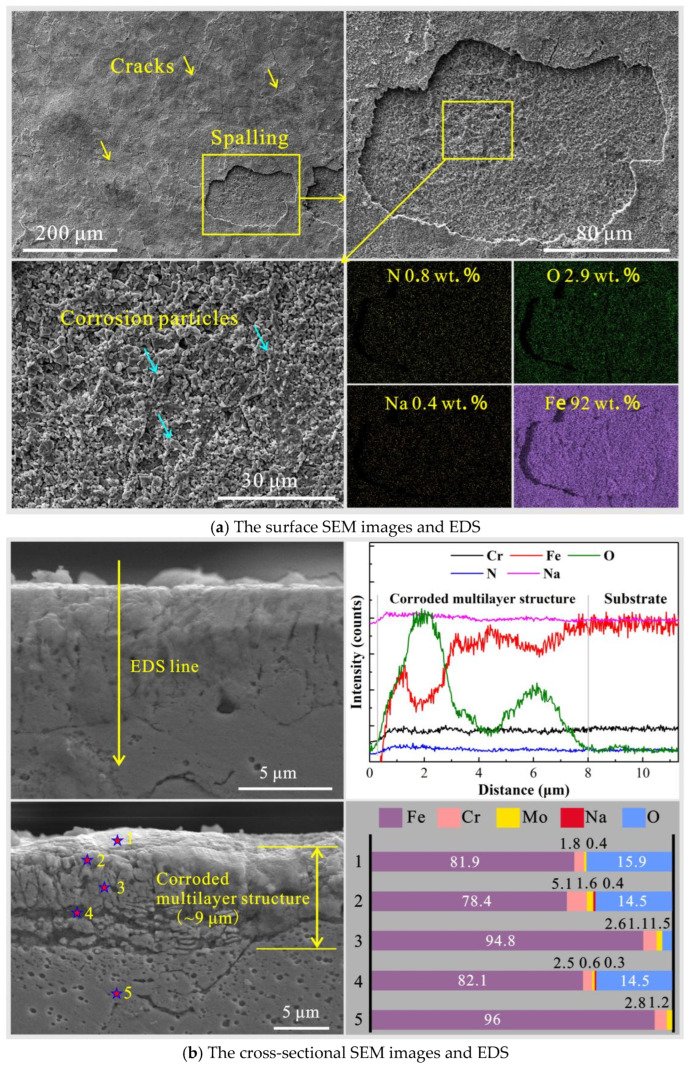
The micro-morphology analysis of the unworn areas of QPQ 590, N = 2 × 10^5^.

Figure 5 and Figure 6 show the characteristics of wear scars and unworn areas of QPQ 590 after 2 × 10^5^ cycles. The 3D morphology shows that the wear scar is slightly higher than the substrate (Figure 5a), and the SEM image finds that there are obvious material fractures at the boundary of the wear scar (Figure 5b). Numerous cracks were found in the exposed nitrided layer, with larger oxide particles agglomerated into flakes with a slightly higher oxygen content than the specific wear scar (Figure 5b). The edge of the wear scar mainly shows delamination and spalling of the material, while the center of the wear scar is a scratch parallel to the fretting direction (Figure 5c). This may be due to the restraint of the material by the larger positive pressure at the center of the wear scar. The material is more likely to peel off at the edge of the wear scar due to the smaller positive pressure, while at the boundary of the wear scar, the material breaks due to insufficient positive pressure. A small amount of N element was found in the center of the wear scar (Figure 5c), and a wear debris accumulation or residual corroded multilayer structure was also found in the cross-section of the wear scar (Figure 5e). This shows that the thicker multilayer still plays a role in resisting wear. The microcracks similar to QPQ 550 were also found in the substrate section of QPQ 590 (Figure 5d).

The unworn area of QPQ 590 showed larger pieces of material spalling, as shown in Figure 6a. The nitrided layer is corroded by liquid sodium, resulting in cracks and then peeling off. The exposed diffusion layer is also corroded by sodium and produces a large number of corrosion products (mainly containing Fe) (Figure 6a). Its cross-section is similar to that of QPQ 550, the multilayer structure is completely broken, and the thickness is reduced to only about 9 μm, as shown in Figure 6b. The oxygen and sodium are mainly concentrated in the corroded multilayer structure surfaces and cracks. This illustrates that QPQ 590 with a thick multilayer structure is more likely to react with sodium, causing cracks and spalling. On the one hand, QPQ 590 will produce some micro-cracks during the nitriding process, which gradually expand in the high-temperature liquid sodium. On the other hand, the difference in thermal expansion coefficient between the thicker multilayer structure and the substrate also makes it more prone to cracking [45].

**Figure 7:**
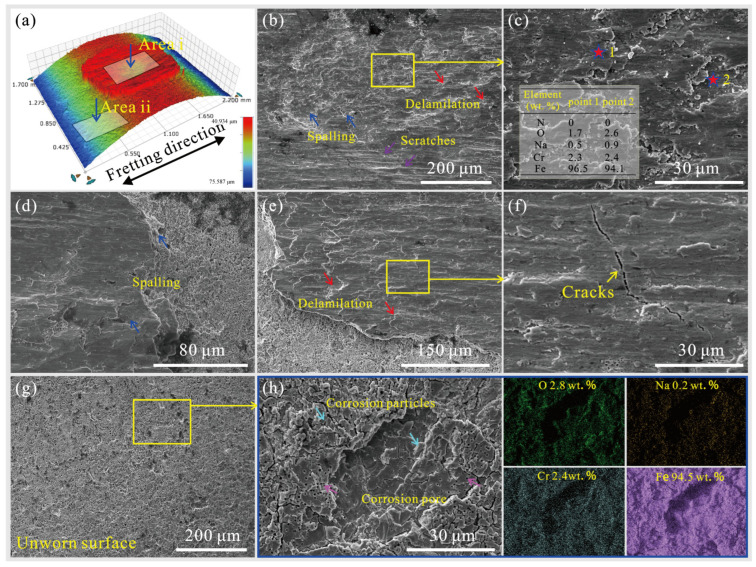
The micro-morphology analysis of QPQ 550, N = 2 × 10^6^: the 3D morphology of the wear scar (**a**); the SEM images and EDS of the worn surface (**b**–**f**), and the unworn surface (**g**,**h**).

**Figure 8:**
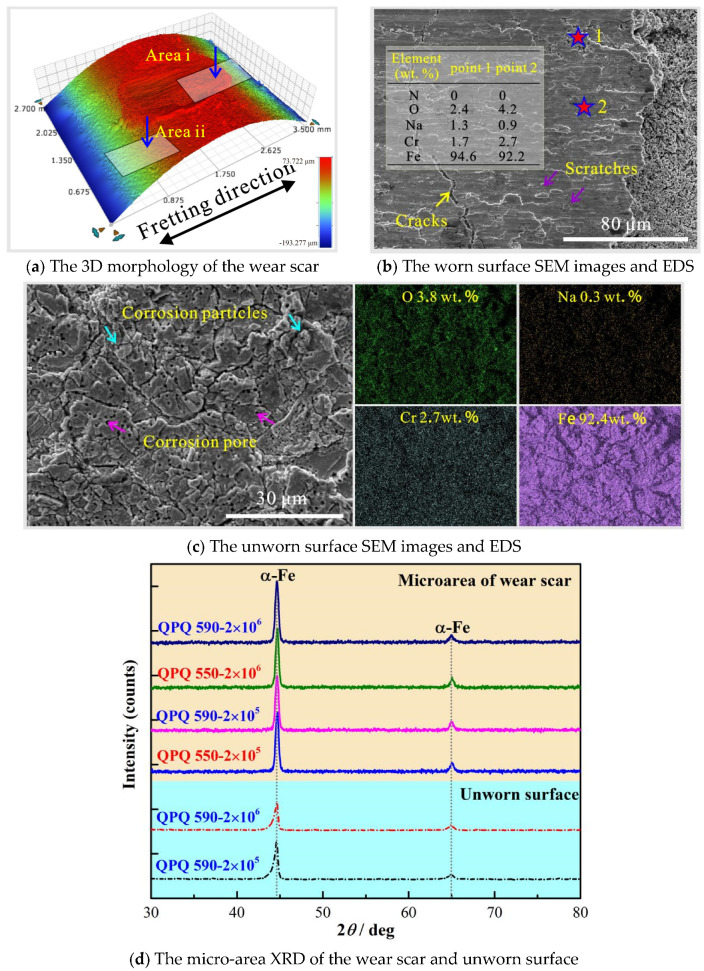
The micro-morphology analysis of QPQ 590 (**a**–**c**), N = 2 × 10^6^; and the XRD of the samples after the test (**d**).

Figure 7 shows the characteristics of the wear scar and the unworn area of QPQ 550 after 2 × 10^6^ cycles. The 3D morphology is similar to Figure 5a, and the wear scar is significantly higher than that of the substrate (Figure 7a). The enlarged images of its marked area are shown in Figure 7b,g. The scratches parallel to the fretting direction and the material spalling were found in the center of the wear scar. The elemental analysis showed that the wear scar centers were mainly Fe, O, Cr, Na, etc. (Figure 7c). Delamination, spalling, cracks, and fractures of the material were found at the edges of the wear scars, as shown in Figure 7d–f. The unworn area shows some cracks, corrosion products, and corrosion micropores, which mainly contain Fe, O, Cr, Na, etc., as shown in Figure 7g,h. It shows that the multilayer structure of QPQ 550 has more serious peeling after sodium corrosion for a longer time. The wear scar retains some residual corroded structure under the effect of positive pressure and surrounding fluid force. After 2 × 10^6^ cycles, the characteristics of the wear scar and unworn area of QPQ 590 are similar to those of QPQ 550, as shown in Figure 8a–c. The wear scar also left a residual corroded structure (Figure 8b). The unworn area was divided into blocks by cracks, some corrosion products were attached to the surface, and a large number of corrosion micropores were found.

The XRD results show that there are only α-Fe peaks in both the wear scar micro-region and the unworn region, as shown in Figure 8d. This shows that the material remaining in the center of the wear scar or transferred from the tribo-pair is all α-Fe. The corrosion structures remaining at the edge of the wear scar and the unworn areas are iron reduced by sodium [16,34]. However, this cannot explain the trace elements such as O and Na detected by the elemental analysis in the previous results. Therefore, QPQ 590, after 2 × 10^6^ cycles, was selected for FIB-TEM testing for further analysis.

**Figure 9:**
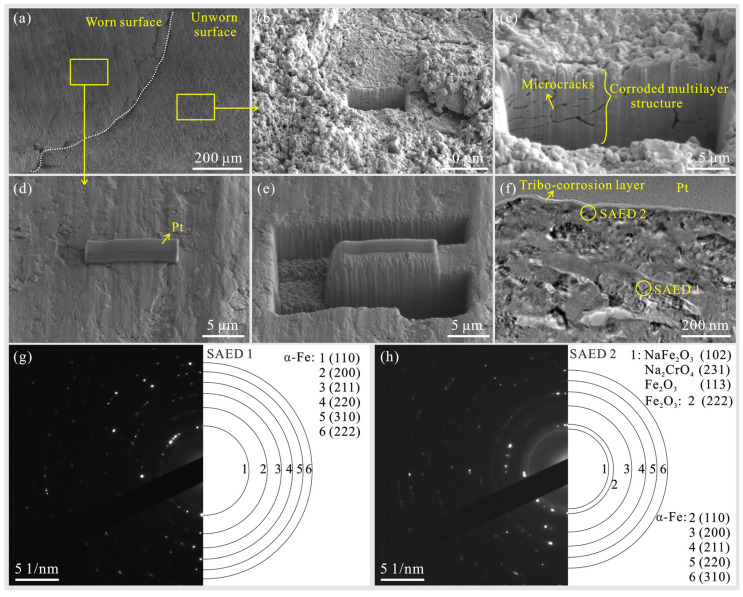
The FIB-TEM analysis of QPQ 590, N = 2 × 10^6^: (**a**) wear scar; (**b**,**c**) unworn surface; (**d**,**e**) worn surface; (**f**) the TEM samples selected from (**e**); (**g**,**h**) the SAED obtained from the marked area in (**f**).

As shown in Figure 9a, the FIB samples were prepared inside and outside the wear scar, respectively. Figure 9b,c shows the unworn area after ion beam cutting. The microcracks can be found inside the corroded multilayer structure, which is consistent with the previous results. The presence of these cracks is not conducive to ion thinning, so the unworn area does not end up with a sample that can be used for TEM testing. The TEM sample preparation at the center of the wear scar is shown in Figure 9d–f. A thin tribo-corrosion layer is found on the surface of the wear scar (Figure 9f). The SAED analysis was performed near the tribo-corrosion layer and deeper (Figure 9f). It can be found that the deeper subsurface layer shows a polycrystalline structure, mainly α-Fe, as shown in Figure 9g. The subsurface layer close to the tribo-corrosion layer also shows a polycrystalline structure, but its diffraction ring shows the presence of some amorphous structure. In addition to α-Fe, these polycrystals also contain some Fe_2_O_3_, NaFe_2_O_3_, and Na_2_CrO_4_, as shown in Figure 9h. This shows that the multilayer structure is mainly α-Fe after tribo-corrosion, and there are also a small number of other corrosion products containing oxygen and sodium, which confirms the previous results.

**Figure 10:**
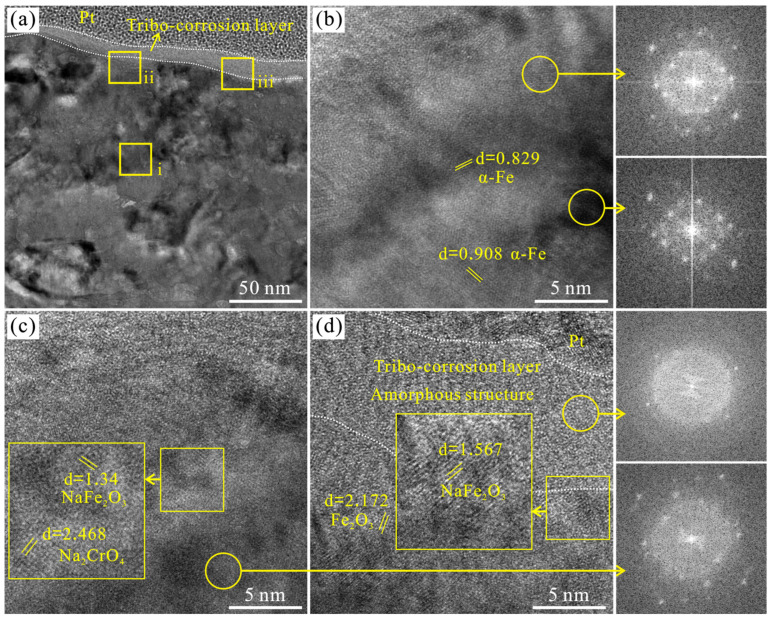
The HRTEM analysis of the subsurface of the worn area of QPQ 590: (**a**) cross-section; (**b**) HRTEM of region i; (**c**) HRTEM of region ii; (**d**) HRTEM of region iii.

Figure 10a further magnifies the subsurface of the wear scar. The tribo-corrosion layer is about 10 nm. The HRTEM of the marked regions in Figure 10a is shown in Figure 10b–d, respectively. It can be found that the deeper subsurface is mainly α-Fe, and its crystal structure is also obtained by the fast Fourier transformation (FFT). Particles such as Fe_2_O_3_, NaFe_2_O_3_, and Na_2_CrO_4_ are found in the tribo-corrosion layer. The FFT of the tribo-corrosion layer comes with an amorphous structure, which may be a chromium-containing oxide [34,35], as shown in Figure 10c,d. It shows that the corrosion product particles composed of Fe, O, Na, and Cr are distributed in the tribo-corrosion layer and cannot be detected by XRD due to the small total amount and small particle size. The formation process of the sodium-containing oxides proceeds as follows: the initial reaction occurs between sodium and the oxide layer, as represented by chemical Equation (1) [49]:

(1) $$8Na+{Fe}_{3}O_{4}\to 3Fe+{4Na}_{2}O$$

By consuming the oxide layer and increasing the oxygen content in the liquid sodium, this step triggers the following chemical reactions (2) [50]:

(2) $$Cr/Fe/Ni/Mo+{Na}_{2}O\to Na+{Na}_{x}{Me}_{y}O_{z}$$

The “Me” refers to alloying elements such as Cr, Fe, Ni, and Mo. This step serves as the primary reaction pathway for the liquid sodium corrosion of materials and the dissolution of alloying elements into sodium. Both an increase in the dissolved oxygen concentration and temperature in the liquid sodium promote this process.

**Figure 11:**
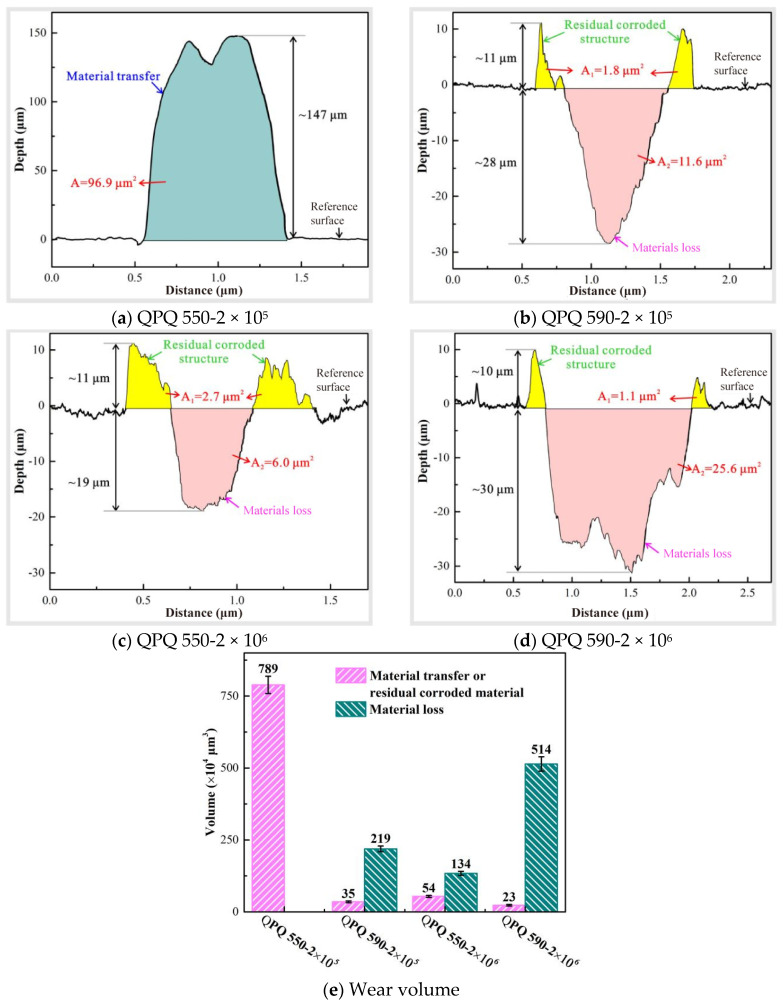
The cross-sectional profiles and the materials transfer or loss volume of the samples.

Figure 11 shows the cross-sectional profiles and wear data of the worn surfaces for different samples. The wear scar cross-sections of QPQ 550 and 590 after 2 × 10^5^ cycles are shown in Figure 11a,b. The QPQ 550 has a material transfer of about 147 μm above the substrate, with an area and volume of about 96.9 μm^2^ and 789 (×10^4^ μm^3^). The thinner multilayer structure is first removed after fretting corrosion, and then, under the effect of high-temperature sodium and frictional heat, tribo-oxidation products accumulate on the worn surface, forming protruding peak. The QPQ 550 did not exist this protruding peak after 2 × 10^6^ cycles (Figure 11c). This shows that this kind of tribo-oxidation products has a certain probability, and it may not exist under the repeated action of the friction force.

The QPQ 590 has a residual corrosion structure of about 11 μm above the substrate (agrees with the result in Figure 5a), and its maximum wear depth below the substrate is about 28 μm. On the one hand, QPQ590 has a thicker multilayer structure (especially a thicker oxide layer), which is more likely to react with sodium and peel off, resulting in a reduction in the plane height of the substrate. On the other hand, the multilayer structures that crack under thermal stress and corrosion are also more easily removed by friction shear. The QPQ 590 has a larger materials loss volume. After 2 × 10^6^ cycles, the maximum material loss volume of QPQ 590 reached 514 (×10^4^ μm^3^).

Both QPQ 550 and 590 have residual corrosion structures about 11 μm higher than the substrate after 2 × 10^6^ cycles (agree with the result in Figure 7a and Figure 8a), as shown in Figure 11c,d. It shows that positive pressure and fluid force can only keep the residual structure up to 11 μm thick. QPQ 550 has a smaller wear depth and material loss volume, probably because the substrate was more wear-resistant than the multilayer structure where QPQ 590 failed.

### 3.3. Discussion

**Figure 12:**
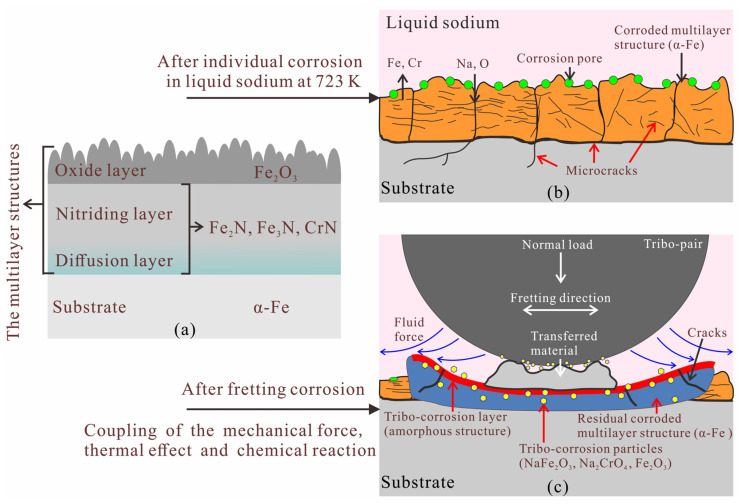
The deterioration and fretting corrosion mechanism of nitrided 2.25Cr-1Mo steel in liquid sodium at 723 K: (**a**) initial multilayer microstructure; (**b**) cross-section after individual corrosion; (**c**) cross-section of the worn area.

Figure 12a shows that QPQ salt bath nitriding can prepare a multilayer structure mainly composed of an oxide layer, nitride layer, and diffusion layer on the surface of 2.25Cr-1Mo steel. The cross-sectional hardness and brittleness of the multilayer structure increase, while the toughness decreases, and it exhibits a different coefficient of thermal expansion than the matrix [42,43]. An increase in the salt bath temperature increases the thickness of the multilayer structure but also causes the material to prematurely microcrack, reducing its properties. The hard multilayer structure can generally improve the wear resistance of the material [51,52,53,54]. The deterioration failure mechanism and fretting corrosion mechanism in high-temperature liquid sodium are shown in Figure 12b,c. After the multilayer structure is individually corroded in high-temperature liquid sodium, the entire multilayer structure is broken, and some surface layers are peeled off. The earliest failure and spalling are the outermost oxide layer, followed by the nitriding layer, and even the diffusion layer will fail and spall for a long time (Figure 4, Figure 6, Figure 7 and Figure 8). During this process, Cr, which has an affinity for sodium, diffuses from the multilayer structure, while Na and O diffuse into the multilayer structure (Figure 12b). Long-term element diffusion will produce a large number of corrosion micropores on the surface (Figure 7 and Figure 8). The diffusion of elements, thermal effect, and the reduction in the toughness of the multilayer structure itself eventually lead to the rupture and failure of the multilayer structure. The microcracks not only become a fast channel for element diffusion to accelerate the corrosion but also divide the residual corrosion structure (which is reduced to iron by sodium) into blocks (Figure 8c and Figure 12b). In addition, cracks can also penetrate the substrate, which is also related to the degeneration of the performance of the substrate in a high-temperature environment.

The friction and corrosion of the multilayer structure in high-temperature liquid sodium are mutually promoting processes. The shear force from friction accelerates material removal at the friction interface [38]. The exposed new material will also corrode faster with sodium [35]. After the multilayer structure fails, the microcracks in it further accelerate material removal and corrosion. However, under the action of positive pressure and fluid forces around the contact point, the corrosion-failed multilayer structure is not easily peeled off, which makes the thicker multilayer structure still play a role in resisting wear. However, the presence of microcracks makes the material easier to remove [54], so the material loss of QPQ 590 is larger. Finally, a thin friction-corrosion layer is formed at the friction interface, and a small number of corrosion products are dispersed in it (Figure 11). The tribo-oxidation products may occur in the substrate and tribo-pair after the failed thinner multilayer structures are removed (Figure 4).

Previous studies have found that QPQ can improve the wear performance of 2.25Cr-1Mo steel in liquid sodium [33], and that is at smaller fretting cycles without complete failure of the multilayer structure. In this study, after a long period of fretting corrosion, the multilayer structure will fail, and its wear performance is poor. If it is in service for a long time, the failure of the multilayer structure will lead to a larger material loss of wear at the fretting corrosion interface. A large amount of spalling will occur in the unworn area, which will not only bring more impurities to the circuit system but also cause the thickness of the heat transfer tube to be reduced. The results suggest that QPQ treatment is potentially suboptimal for nuclear-grade 2.25Cr-1Mo steel under 723 K sodium conditions, and the surface modification technology of 2.25Cr-1Mo steel needs to be further studied.

## 4. Conclusions

In this study, the deterioration failure and tribo-corrosion mechanism of nitrided 2.25Cr-1Mo steel in liquid sodium at 723 K have been investigated. The conclusions are summarized as follows:

(a) The multilayer structures fabricated by salt bath nitriding significantly enhance the cross-sectional hardness and tribological performance of the substrate. Notably, the QPQ 550 treatment, characterized by a thinner compound layer, exhibited superior wear resistance during extended testing compared to the thicker QPQ 590 variant, primarily due to its higher resistance to crack initiation.
(b) Prolonged exposure to flowing liquid sodium induces the failure of these nitrided layers, governed by the synergistic effects of tribological removal, chemical corrosion, and thermal acceleration. High-resolution TEM (HRTEM) analysis reveals that the near-surface region evolves into a nanometric tribocorrosive layer. This layer is composed of complex reaction products, including Fe_2_O_3_, NaFe_2_O_3_, and Na_2_CrO_4_, indicating active chemical interactions between the matrix elements and the sodium environment.
(c) While a higher nitriding temperature (QPQ 590) promotes the formation of a thicker modified layer, it concurrently leads to the premature generation of micro-cracks within the compound layer. Under the combined effects of thermal stress, mechanical loading, and chemical attack, these micro-cracks serve as preferential channels for sodium penetration. This accelerates the corrosion-wear process and ultimately results in inferior long-term performance. Therefore, strict control over the modified layer thickness is essential to suppress micro-crack formation and ensure durability in sodium environments.

## Data Availability

The datasets presented in this article are not readily available because the data are part of an ongoing study. Requests to access the datasets should be directed to corresponding authors.
